# Coupling Coordination Relationship and Driving Mechanism between Urbanization and Ecosystem Service Value in Large Regions: A Case Study of Urban Agglomeration in Yellow River Basin, China

**DOI:** 10.3390/ijerph18157836

**Published:** 2021-07-23

**Authors:** Kaili Zhang, Tan Liu, Rongrong Feng, Zhicheng Zhang, Kang Liu

**Affiliations:** 1 College of Urban and Environmental Sciences, Northwest University, Xi’an 710127, China; zhangkaili@stumail.nwu.edu.cn (K.Z.); rongzi0912@foxmail.com (R.F.); zhangzhicheng1@stumail.nwu.edu.cn (Z.Z.); 2 School of Economics and Management, Northwest University, Xi’an 710127, China; liutan@stumail.nwu.edu.cn; 3 National Forestry and Grassland Administration Urban Forest Ecosystem Research Station, Xi’an 710127, China

**Keywords:** coupling coordination degree model, spatial autocorrelation statistical model, geographical detector, GWR model

## Abstract

Mastering the coupling and coordination relationship and driving mechanism of urbanization and ecosystem service value (ESV) is of great significance to ecological protection and regional sustainable development. In this paper, the coupling coordination model, geographic detector and GWR model are used to analyze the spatio-temporal coupling interaction between urbanization and ESV and the spatial differentiation characteristics of influencing factors from 1995 to 2018. The results of the study are as follows: (1) During the study period, cities in the Yellow River Basin experienced accelerated urban expansion, and the ESV of forests, water and wetlands increased, which offset the reduction in ESV due to the expansion of construction land and farmland and grassland. (2) The degree of coupling and coordination between the two gradually improved, but the overall situation showed a low-level coupling and coordination process. Mild coupling coordination gradually increased, reaching an increase of 38.10%; severe imbalance types tended to disappear, decreasing by 52.38%, and coupling subtypes developed from lagging urbanization to ESV backward types. The high-value areas of the coupling coordination degree are distributed in the high-value areas of ESV in the north of the upper reaches, and the low-value areas are distributed in the cities of Henan and Shandong with high urbanization levels in the downstream and most resource-based cities in the middle reaches. (3) In addition, the spatial intensity of the effect of each dominant factor on the degree of coupling coordination is different. Economic growth, technological development, environmental regulations and the proportion of forest land have positive and belt-shaped alienation characteristics for the coupling and coordination of the two, and infrastructure and temperature show negative driving characteristics. Therefore, the coupling and coordination relationship between ESV and urbanization should be clarified to help future urban planning. On the basis of determining the regional environmental carrying capacity and the adjustment direction of the rational planning of land resources, the impact of urban barriers formed by administrative boundaries and natural geographical conditions on the development of urban agglomerations should be broken to achieve the overall high-quality and coordinated development of the basin.

## 1. Introduction

Ecosystem services (ESs) are defined as the products or benefits that people obtain directly or indirectly through the structure, functions and processes of ecosystems [1,2]. The Millennium Ecosystem Assessment divides them into four categories based on the links between the ecological environment, society and human well-being, namely, the supply, regulation, support and cultural services that are vital to maintaining life and protecting the integrity of the ecosystem [3,4]. However, with population growth and economic development, the land cover on the earth’s surface has undergone tremendous changes. Agricultural landscapes and human settlements account for 75% of the Earth’s ice-free surface, which has a huge impact on ecosystem functions and processes [5]. The 2020 IPBES Biodiversity and Ecosystem Services Global Assessment Analysis found that although the supply services of ecosystems have increased, the regulation services and biodiversity have declined, indicating that the ability of ecosystems to maintain their contributions to humans is being compromised [6]. Therefore, enhancing the understanding and management of the relationship between man and nature is of great significance to regional ecological protection and sustainable development [7].

Ecosystem service value (ESV) provides an intuitive perspective from which environmental managers and the public can directly understand the potential benefits of the ecosystem [8]. Costanza used the monetary evaluation method to calculate the global ecological capital and the value of ESs, which greatly promoted the evaluation of global ecosystem services [2]. This method has been widely adopted, due to its advantages, such as ease of use, high comparability of results and comprehensive evaluation range [9,10,11,12]. However, natural contributions, such as energy supply, water quality regulation and coastal risk reduction, are not evenly distributed around the world, and human needs vary from place to place. Therefore, it is necessary to consider the needs of different regions [6]. For example, combined with the results of Costanza’s research, Xie [13] established a Chinese ESV assessment system based on expert knowledge, and revised the equivalent factors of Chinese land landscape types. In addition, some scholars further revised the ESV evaluation methods and equivalent factors based on factors such as net primary productivity (NPP), resource consumption and consumption levels [14,15], and established a series of models and methods, for example, using InVEST for the comprehensive valuation of ESs and trade-offs [16], using Artificial Intelligence for Ecosystem Services (ARIES) [17], using the SolVES model for social value evaluation [18], using the MIMES model for multi-scale comprehensive evaluation [17], and using the ES value to evaluate ESs in a specific region [19]. Among them, the InVEST, ARIES and MINMES models have high versatility and can be applied at global, watershed and landscape scales through simplified algorithms [19].

Previous studies have confirmed that ESV can partially reflect the interaction and co-evolution process between land use and ecosystems and further strengthen the understanding of the relationship between land use and socio-economic development [20,21,22]. For example, some scholars have analyzed the impact of landscape pattern changes on the value of ecosystem services by linking the dynamic changes in land use with ESV, and further provide countermeasures for future land planning and energy use [23,24]; Long et al. combined ecosystem services and land-use changes, proposed the construction of an ecological compensation mechanism, clarified important ecological function areas and protected areas, and implemented reasonable spatial control [21]. As a bridge that couples natural and social processes, ESs provide new theoretical support for studying the coupling and coordination relationship between humans and natural systems [20].

At present, 60% of the global ecosystem functions are being or have been degraded, which is mainly due to the interference of human activities [3]. Especially for urban areas where human activities are the most concentrated and frequent, they promote changes in ESs from many aspects [25,26]. Urbanization is a multi-dimensional development process of the population, economy, society and other factors. Its essence is a comprehensive process that combines inward agglomeration and outward expansion [27], and its characteristics are often expressed in the agglomeration and transfer of population, economic development, urban land expansion, changes in human lifestyles and consumption levels [26,27]. These are also the main driving factors for land change and biodiversity loss, and are an important part of global environmental change [28].

There are complex interactions between urbanization and ecosystems, and these interactions have pressures and constraints [29]. On the one hand, economic development, population agglomeration, resource consumption and the expansion of construction land in the process of urbanization have led to the fragmentation of the landscape [30] and continuous degradation of ESs [31] (decline in biological and ecological diversity, degradation of ecosystems, soil erosion and desertification). The fragmented landscape pattern weakens the role of the ecosystem as a buffer between humans and the natural environment [32]. Humans and landscapes co-exist in an interactive system. Changes in landscape patterns increase human demand for ES, and cause various environmental problems [33]. Many studies have shown that areas with a higher level of urbanization have greater demand for ESs [8]. On the other hand, some scholars in China have found that economic development can improve ecosystem service capabilities through scale effects, optimizing industrial structure and improving environmental awareness and management level [27]. Therefore, in order to promote a coordinated relationship between economic development and ecological protection, it is necessary to analyze the coupling–interaction relationship between the urbanization process and the ecosystem [8,25].

The ecosystem and human society should be in a dynamic and balanced evolutionary process. If the pursuit of urbanization is excessive, it may cause the socio-economic pressure to exceed the maximum threshold of the ecosystem’s carrying capacity, thereby causing the collapse of the ecosystem and threatening human survival and development [34]. Therefore, how to quantitatively evaluate the pressure of the ecosystem in the process of urbanization and how to coordinate the relationship between urbanization and ESV are important issues in promoting regional sustainable development.

Many scholars are working to explore the relationship between urbanization and ES. Most of the studies are based on a single factor and use mathematical, statistical or spatial analysis models to explore the correlation between the level of urbanization and ES, such as negative correlation [35], positive correlation [36] and “inverted U-shaped” [25,27]. However, this type of research lacks an analysis of the degree of coupling between ESV and integrated urbanization, especially the differentiation rules of spatial coupling and coordination characteristics [37]. A small number of scholars have conducted research on the coupling and coordination interaction between urbanization and ESV, but these studies focus more on the spatiotemporal characteristics of the coupling and coordination degree [25,27], without further in-depth analysis of the factors that affect the coupling and coordination degree of the two. At the same time, most of the research scales they choose are specific river basins, provinces, cities or counties, while ignoring the differences in the coupling interaction between urbanization and ESV in the inner space of large regional urban agglomerations and the coupling and coordination driving mechanism. Therefore, it is of great significance to establish an effective modeling method to clearly quantify the spatiotemporal coupling relationship between integrated urbanization and ESV [38], and to deeply analyze the driving mechanism that affects the degree of coupling and coordination.

The process of urbanization usually leads to changes in the environment and society of a certain area, and these changes are more obvious in urban agglomerations, due to the rapid growth of population, economy and space, and huge demand for land and natural resources [27,28,37,39,40]. Urban agglomerations are the spatial entities that promote modern urbanization in China [41]. The Yellow River Basin spans the three major urban areas of China’s east, middle and west. As the main part of China’s new urbanization, it is a brand-new regional unit that promotes economic development and participates in global competition [42,43]. However, due to the fragile ecological environment and lagging economic development, it has become a key and difficult area for national ecological security and economic and social development [44]. In 2019, the ecological protection and high-quality development of the Yellow River Basin became a national strategy; thus, it has long-term strategic significance and regional representativeness for research in this region.

Therefore, this paper takes the urban agglomerations in the Yellow River Basin in China as an example to explore the coupling interaction and driving mechanism between urbanization and ESV in large regions, and raises the following questions: For the cities flowing through the Yellow River Basin, how does urbanization affect ES? Has the coordination and interaction between ESs and urbanization been realized? At the same time, for the interior of the region, is there spatial heterogeneity among cities, and what are the differences in their respective influencing factors? In view of this, the innovation of this paper lies in the scientific evaluation of the coordination degree of urban ecosystem services and urbanization in the Yellow River Basin, and the use of geographic detectors and the GWR model embedded with geographic location information to further investigate the temporal and spatial differentiation characteristics of its influencing factors. It also proposes different strategies for different types of regional coupling and coordination. Based on different types of coupling and coordination, combined with ecological function zoning for spatial management and control, this will provide a basis for biodiversity conservation and new urbanization in the Yellow River Basin.

## 2. Materials and Methods

### 2.1. Study Area

**Figure 1:**
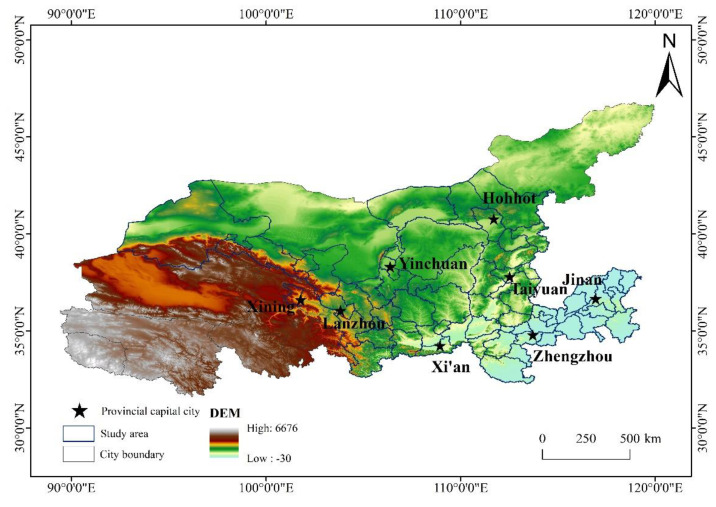
Geographical location of the study area, Yellow River Basin, China.

The Yellow River Basin is located at 32° N~42° N, 96° E~119° E, with a length of 5464 km and an area of 752,442 km^2^. The terrain in the basin is high in the west and low in the east, and the landforms are extremely different. In addition, the climate in the region is significantly different. Specifically, the southeast, central and northwestern regions belong to the semi-humid, semi-arid and arid climates, respectively [45]. The study area as a whole presents a significant pattern of physical geographic differentiation, which also determines the significant differences in the ability of each region to provide ES. The urban agglomerations in the Yellow River Basin are important agricultural areas, industrial belts and ecological security barriers in China. As a multi-level network system, there are regional differences in their natural environment and socio-economic development [46]. However, in recent years, the continuous and rapid growth of urbanization in the Yellow River Basin has led to a substantial increase in the demand for ES, which has put tremendous pressure on the ecological environment and space resources [47], considering the complexity of regional ecology, economy and political management, and using it as a demonstration of methods to solve environmental degradation and regional development imbalances. This will provide a reference for exploring the new model of optimized and coordinated development under the constraints of the ecological environment. In the comprehensive cross-administrative study, the boundaries of the Yellow River Basin are divided differently [48]. According to the “Administrative Divisions of the People’s Republic of China” (http://www.gov.cn/test/2005-06/15/content_18253.htm, accessed on 14 July 2021), we selected 63 prefecture level cities flowing through the Yellow River Basin as the study area. Considering the spatial proximity effect of urban agglomerations, the cities we referred to are in a broad sense, including the central urban area and its surrounding suburbs and rural areas. We regarded them as a whole connected spatial economic and social entity, which is more conducive to us to propose spatial control measures and sustainable development directions on a macro scale (Figure 1).

### 2.2. Data Sources

The research data include land use data, meteorological data, digital elevation model (DEM) data and socio-economic statistics in the Yellow River Basin. Among them, the land use data were obtained from the “China Land Cover Raster Data (30 m × 30 m)” of the Resource and Environmental Science Data Center of the Chinese Academy of Sciences; the annual temperature and precipitation data were obtained from the platform of the China Meteorological Administration; and the socio-economic data were obtained from the 1995–2018 China Statistical Yearbook, China Urban Statistical Yearbook, China Urban and Rural Construction Statistical Yearbook, China Environment Statistical Yearbook, China Energy Statistical Yearbook and Statistical Yearbooks of related provinces (regions) and cities in the Yellow River Basin. In addition, the individual indicators were missing, and the interpolation method and the mean method were used to fill them in; based on the data of adjacent years, we used the gray correlation degree to determine the final data.

### 2.3. Study Methods

#### 2.3.1. Ecosystem Service Assessment

According to the characteristics of land resources in the study area and research objectives, combined with the Land Use Classification System of China and field survey, the land use data were re-divided into 7 categories using ArcGIS 10.4, namely, cultivated land, forest land, grassland, water area, wetland, construction land and unused land [25,27,49]. Among them, there were forest land (natural forests and artificial forests with a canopy density greater than 30%); shrubs (dwarf woodland and shrubland with a canopy density greater than 40% and height less than 2 m); sparse woodland (woodland with a canopy density of 10–30%); other woodlands (including various nurseries, orchards, tea plantations and tropical gardens); water bodies, including rivers (refers to naturally formed or artificially excavated rivers and land below the annual water level of the main trunk), lakes, permanent glaciers and snow; wetlands, including reservoirs, ponds and beaches; and unused land, including sandy land, desert and bare land.

In order to calculate the ESV of the Yellow River Basin, this paper chose the monetary method, mainly referring to Costanza’s ESV equivalent factor method, Xie’s questionnaire-based ESV equivalent factor table and regional correction coefficients of China [2,10,13,50]. Many scholars believe that natural grain yield is 1/7 of the actual value [13,25,50]. Based on the average prices of the three main grains, this paper took the average net profit of the farmland ecosystem, excluding human input costs as the standard equivalent factor, and used the ratio of the unit grain output in the cities of the Yellow River Basin to the national grain output per unit area in the same period as the correction coefficient [51]. Furthermore, taking into account the inflation, the grain consumer price index was used to revise grain prices. In the end, the economic value of an equivalent factor was determined and multiplied by the unit area of different land use types. In addition, considering that supporting services were the intermediate, they were not included in the final total ESV to avoid double counting. The calculation formula of ESV is as follows:

(1) $$\mathrm{ESV}=\sum S_{i}\times {\mathrm{VC}}_{i}\;\delta =1/7\times \sum_{1}^{z}R_{z}N_{z}N_{z}/3\times (\alpha /\beta )\times \lambda$$

In Formula (1), ESV is the total value of ecosystem services (in CNY); S_i_ is the area of the i-th ecosystem (in ha); VC_i_ is the service value of each unit area of ecosystem i; δ is the equivalent value of a standard unit in the region; R_z_, M_z_ and N_z_ represent the prices of three major food crops, wheat, corn and rice, respectively; α and β, respectively, represent the grain output per unit area of the cities in the study area and the national grain output per unit area in the same period; and λ is the resident food consumption price index.

#### 2.3.2. Establishment of Urbanization Index System

This paper drew on existing research results, while considering the scientificity, validity and availability of the data, as well as a comprehensive assessment of urbanization. We selected a comprehensive socio-economic data set within prefecture-level cities. The entropy method was used to assign weights to various indicators, and finally, the comprehensive evaluation index for urbanization in the Yellow River Basin was determined (Table 1).

#### 2.3.3. Variable Selection of Driving Factors

In order to explore the main influencing factors of the coordinated development of ESV and urbanization, this article took 2018 as an example, combined with the actual situation of the coordinated development of ESV and urbanization in cities in the Yellow River Basin. We referred to the relevant literature [16,25,52] and interviewed 6 experts in the ecology and geography research fields. After comprehensive consideration, we finally selected 12 indicators from three major aspects of social, economic and natural factors as detection factors (Table 2) and conducted empirical research on the driving mechanism of the coordinated development of ESV and urbanization by using geographic detector analysis methods. In ArcGIS 10.4, the natural breakpoint method was used to classify each element, and the influence of each element on the degree of coupling and coordination was calculated. Based on the detection results, combining the detection value of the factor and the significance test realizes the identification and construction of the dominant factor.

#### 2.3.4. Data Standardization and Index Empowerment

In order to eliminate the shielding effect among the data and the influence of the magnitude and dimension difference of each index on the calculation result, it is necessary to standardize each index to reduce the interference of random factors. This paper used the range standardization method to standardize the original data, and its formula is as follows:

x_mj_ = (x_mj_ − min(x_mj_)/(max(x_mj_) − min(x_mj_)) (+) x_mj_ = (max(x_mj_) − x_mj_)/(max(x_mj_) − min(x_mj_)) (−) (2)

In Formula (2), X_mj_ is the standardized value of the j-th index of each system. When m is E, it represents the ESV system; when m is U, it represents the urbanization system. x_mj_ represents the original value of each system. max (x_m__j_) and min(x_m__j_) are the maximum and minimum values of the j-th index in each system, respectively.

In order to reduce the influence of individual subjectivity on indicator weights, we adopted a more objective entropy method to determine indicator weights. This method is used to comprehensively evaluate the urbanization system and ESV by assigning the weight of each indicator. The calculation formula of the entropy method is as follows:

(3) $$\lambda_{U,E}=\sum_{j=1}^{n}W_{\mathrm{ij}}\times X_{\mathrm{ij}}$$

In Formula (3), W_mj_ represents the weight of each indicator, λ_U_ represents the comprehensive evaluation index of the urbanization system and λ_E_ represents the comprehensive evaluation index of the ESV system.

#### 2.3.5. Coupling and Coordination Model

The coupling and coordination model can quantify the coordination degree of the interaction and coupling between the ESV and the urbanization system, and can understand the problems in the development process in a more detailed and clear manner. This paper refers to the research of Tang [55] and introduces the Euclidean distance to measure the coupling degree of the two systems in order to examine their coordination. The model emphasizes the definition of the ideal state, that is, the deviation between the actual value of the evaluation variable and the ideal value. Assuming that the ideal coordination state of urbanization and ESV is W’, when the system is in ideal coordination, the two systems pull each other and are in the same development state. According to the ideal coordination state, taking the development degree of the two systems as the evaluation variable, the ideal value is equal to the actual value of the development degree of the other system. Let w_1t_, w_1t_’, w_2t_ and w_2t_’ represent the actual and ideal values of the development degree of the urbanization system and the ecological environment system in year t; then, there is (w_1t_ ∙ $w_{2t}^{\prime }$)^T^ = (w_2t_ ∙ $w_{it}^{\prime }$)^T^. The calculation formula of distance coordination degree is as follows [55]:

(4) $$C_{t}={\left[1-{\left(\sum_{1}^{2}(w_{\mathrm{it}}-w_{it}^{\prime })/\sum_{1}^{2}Q^{2}\right)}^{1/2}\right]}^{k}$$

Assuming that the two systems are equally important, take Q^1^ = Q^2^ = 1, k as the adjustment coefficient; generally, take k = 1, where C_t_ represents the coordination degree of the system in the t-th year. The larger the C_t_ value, the closer the distance between the actual coordination state and the ideal coordination state of the system; the value range of C_t_ is between 0 and 1.

It is difficult to explain the coordination level between systems only by measuring and analyzing the degree of coupling between systems. In order to measure the degree of coordination between the two systems more clearly and accurately, it is necessary to further construct a model of the degree of coupling and coordination. The coupling coordination degree can not only truly reflect the coupling level between the systems, but also show the coordination degree of the interaction between the two systems. The calculation formula is as follows:

D_it_ = (C_it_ × T_it_)^1/2^ T_it_ = (αλ_E_ + βλ_U_)^1/2^ (5)

In Formula (5), D_it_ is the degree of coupling coordination, and D_it_ ∈ [0, 1]. The degree of coupling and coordination of the two systems increases as the D_it_ value increases. T_it_ is the comprehensive evaluation index; α and β are the contribution rates of ESV and the urbanization level system, respectively. According to related research [27], considering that the contribution rates of the two are equal, take α = β = 0.5.

According to the coupling coordination degree value, the coupling coordination level of the two subsystems was divided into 4 major categories and 12 subcategories. The specific categories and corresponding numerical values are as follows (Table 3).

#### 2.3.6. Spatial Correlation Model

As there might be a spatial spillover effect between ESs and urbanization at the urban level in the Yellow River Basin, this paper used Moran’s I to explore whether there is a certain law or correlation between the coupling and coordination of the two in space and region. The formula is as follows:

(6) $$I=(\sum_{i=1}^{n}w_{\mathrm{ij}}(x_{i}-x)(x_{j}-x))/(S^{2}\sum_{i=1}^{n}\sum_{j=1}^{n}w_{\mathrm{ij}})$$

In Formula (6), w_ij_ is the (i,j) element of the spatial weight matrix, and the value range of Moran’s I is [−1, 1]. When Moran’s I > 0, it indicates a positive correlation; that is, the high value of the coupling coordination degree is adjacent to the high value, and the low value is adjacent to the low value. When Moran’s I < 0, it indicates a negative correlation; that is, the high value of the coupling coordination degree is adjacent to the low value. When Moran’s I = 0, it means no correlation; that is, the coupling coordination degree is randomly distributed in space, and there is no spatial correlation. The greater the absolute value of Moran’s I, the closer the spatial relationship.

In order to perform the test more rigorously, the asymptotic distribution of Moran’s I must be derived. Moran’s I values were transformed to Z-scores as follows [56]:

Z = (I − E(I))/(VAR(I))^1/2^ (7)

where E(I) is the expectation of Moran’s I under the null hypothesis that there is no spatial dependence, and VAR (I) is the variance of Moran’s I. The Z-scores indicate the spatial cluster intensity. Z-scores were used to test the significance of any spatial autocorrelation; the higher the Z-score value, the more significant the spatial agglomeration effect of Moran’s Index.

#### 2.3.7. Geographical Detector Method

This study mainly used the factor detection function of the geo-detector. Through the screening of the primary factors, the invalid factors and the dominant factors were identified, and several factors with a high degree of explanation were incorporated into the model to avoid possible multicollinearity problems to the greatest extent. The factor detector can detect the extent to which factor X explains the spatial differentiation of dependent variable Y, which is measured by the q value [57].

(8) $$q=1-(\sum_{h=1}^{L}N_{h}\partial_{h}^{2}/N\partial^{2})\times \partial_{h}^{2}$$

where h and L are the stratification of variable Y or factor X; N_h_ and N are the number of units in the stratification h and the whole area, respectively; $\partial_{h}^{2}$ and ∂^2^ are the variance of the Y values of the h stratification and the whole region, respectively; the value range of q is [0, 1]. The larger the q value, the stronger the explanatory power of the independent variable X to the attribute Y, and vice versa [57].

#### 2.3.8. Ordinary Least Squares (OLS)

In order to explore the driving factors that affect the coupling degree of urbanization and ESV, the least square method (OLS) is used. The OLS model is the basic model of spatial modeling and the benchmark of analysis. In spatial modeling with OLS, it is assumed that the coefficients or parameters of the statistical model are constant relative to the position, and the influence of the change of spatial position is not considered, which is considered the weakness of this method in spatial modeling [58]. The formula is as follows:

y_i_ = β_0_ + ∑_i_β_i_x + ε_i_ (9)

where β_0_ is a constant term; β_1_ is the regression coefficient; and ε_i_ is the random error term.

#### 2.3.9. GWR Model

Observed samples at the city level have differences in geographic location, and the coefficient β of the assumed regression model in the classical spatial measurement model is a constant, so it cannot reflect the changes in the regression coefficients of the explanatory variables in different regions. The spatial variable parameter regression model—Geographically Weighted Regression Model (GWR model), proposed by Fortheringham [59]—can embed the geographic location of the regression unit into the regression parameters, thereby making up for the shortcomings of the traditional measurement model. The GWR model formula is shown in Formula (10).

(10) $$Y_{i}=\beta_{0}(\mu_{i},v_{i})+\sum_{k=1}^{p}\beta_{k}(\mu_{i},v_{i})X_{\mathrm{ik}}+\varepsilon_{i}$$

In Formula (10), (μ_i_,v_i_) is the coordinates of the i-th sample point; β_k_(μ_i_,v_i_) is the k-th (independent variable) regression parameter on the i-th sample point, which is a function of the geographic location; X_ik_ is the value of the k-th independent variable at the position (μ_i_,v_i_); ε_i_ is the random error of the i-th sample point.

## 3. Results

### 3.1. Urbanization

**Figure 2:**
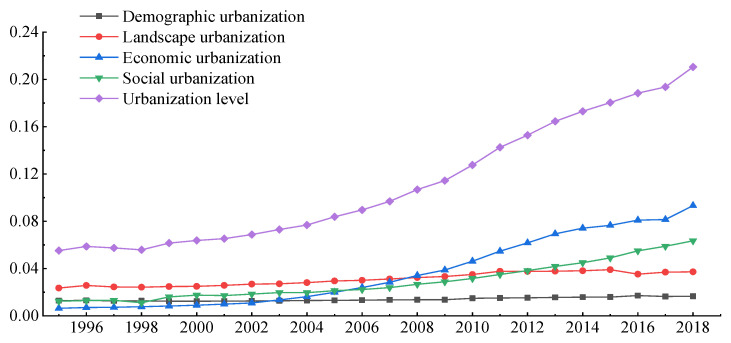
The urbanization level change in the Yellow River Basin (China) from 1995 to 2018.

In general, the comprehensive level of urbanization in the Yellow River Basin continued to improve from 1995 to 2018, with an overall increase of 3.82 times (Figure 2). It maintained slow growth from 1995 to 2005. From 2005 to 2018, urbanization entered a period of rapid development, and the rate of urbanization increased greatly. The development of economic urbanization had the greatest impact on the improvement of the comprehensive urbanization level. Economic urbanization represents an increase in the proportion of urbanization and plays an increasingly important role in promoting the process of urbanization. The urbanization of the population and space gradually increased, and its impact on comprehensive urbanization was relatively stable. Although the population urbanization curve shows an upward trend, it maintains a low level, reflecting its weak influence on the overall urbanization level. The results show that the urbanization process of the urban agglomeration in the Yellow River Basin changed from the initial urbanization stage to an intermediate stage characterized by social urbanization and economic development. This stage depends on rapid economic development, the increasing material base of people, and the improvement of living standards. Various measures must be adopted to greatly improve the level of social urbanization in order to promote the development of advanced stages of urbanization.

### 3.2. Land Use Changes in the Yellow River Basin

**Figure 3:**
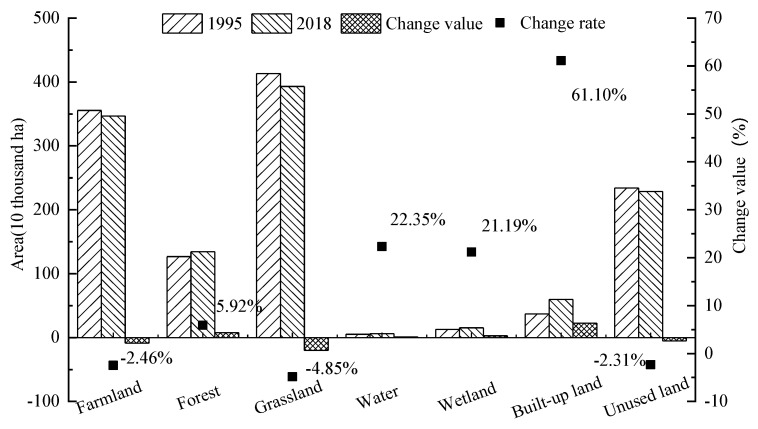
Urban land-use changes in the Yellow River Basin (China) in 1995 and 2018.

From 1995 to 2018, the area of different land types in the Yellow River Basin underwent major changes. Different land types changed significantly, but the overall contribution was relatively stable. The order of the contribution of each land type from high to low is as follows: grassland > farmland > construction land > unused land > forest > wetland > water area (Figure 3). From 1995 to 2018, the area of different types of land use in the cities of the Yellow River Basin changed significantly: farmland, grassland and unused land use all showed a downward trend, with decreases of 2.46% (8.74 × 10^5^ ha), 4.85% (20.04 × 10^5^ ha) and 2.31% (5.39 × 10^5^ ha), respectively, while the areas of the other four land ecosystems increased. Forest land increased by 5.92% (7.50 × 10^5^ ha), water increased by 22.35% (1.14 × 10^5^ ha), and wetland increased by 21.19% (2.68 × 10^5^ ha); the largest increase in was in construction land, by 61.10% (22.66 × 10^5^ ha).

### 3.3. ESV Changes in the Yellow River Basin

#### 3.3.1. ESV Changes Based on Time Scale

**Figure 4:**
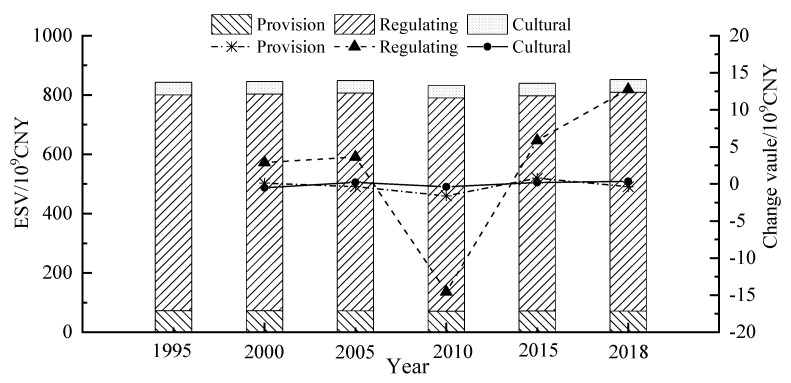
The value of each ecosystem service and change in respective years (1995 to 2018) in the Yellow River Basin, China.

The ESV changes in different land use types and different ESs in the Yellow River Basin are shown in Table 3 and Figure 4.

From 1980 to 2018, the ESV of various land-use types in the cities of the Yellow River Basin changed greatly. The total value increased from CNY 843.14 × 10^9^ to CNY 852.23 × 10^9^, i.e., an increase of CNY 9.09 × 10^9^ (Table 4), which shows that the ESV greatly improved during this period. The value contribution rate of each land type from high to low is as follows: grassland > forest > farmland > wetland > water > unused land. Among them, the value of grassland, farmland and unused land declined, and the value of grassland incurred the most serious loss, which was CNY 20.310 billion, with a change rate of −4.85%, followed by farmland, which decreased by CNY 3700 billion, with a change rate of −2.46%. Unused land decreased by CNY 180 million, with a change rate of −2.31%. However, the values of forests, waters and wetlands increased to varying degrees. Among them, wetlands had the highest increase of CNY 21.160 billion, with a change rate of 21.19%. Forests and waters increased by CNY 9.02 and 3.100 billion, respectively, with a change rate of 5.92% and 22.35%. The change in each kind of land ecosystem was directly proportional to the change in land use area. Although the area of wetlands and waters was small, their high-value coefficients also caused drastic fluctuations in the total value and structure of the ES of the entire land, which offset the overall decline in ESV, due to the reduction in grassland and farmland. Overall, the total amount of ESV increased significantly from 1995 to 2005, decreased significantly from 2005 to 2015 and increased significantly from 2015 to 2018.

Considering that supporting services are intermediate services, in order to avoid the double counting of ES, this paper does not include support services in the total value of ESV. As shown in Figure 3, the value of various ESs in the Yellow River Basin from high to low is as follows: regulating services > provisioning services > cultural services. This indicates that the ESs of cities in the Yellow River Basin are mainly regulating services. From 1995 to 2018, the value of various service types changed to varying degrees. Among them, regulating services showed an increase, with a change rate of 1.46%, and provisioning services and cultural services both showed a decrease, with a change rate of −1.96% and −0.28%. In general, the values of individual ESV subtypes did not change much, and their trends were quite consistent with those of ESV in general. Although the values of various ESV subtypes fluctuated, these fluctuations did not fundamentally change the structure of ESV. Regulating services contributed more than half of the ESV value (86.31–86.64%), followed by provisioning services (8.35–8.61%) and cultural services (5.00–5.08%). The level of change of the ESV subtype value is the same as the level of the total value.

#### 3.3.2. ESV Changes on the Spatial Scale

**Figure 5:**
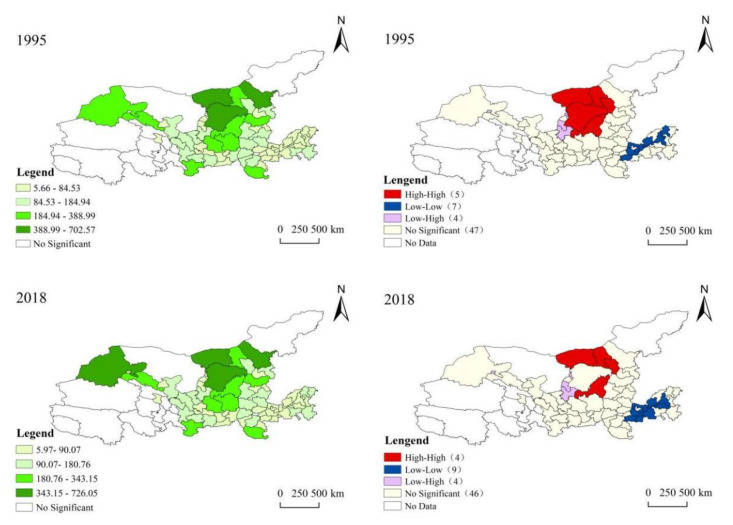
Spatial distribution of urban ecosystem service value in the Yellow River Basin (China) in 1995 and 2018.

We further analyzed the spatial heterogeneity of ESV, considered the mutual influence of neighboring cities, measured the spatial autocorrelation of ESV to determine the degree of spatial agglomeration of ESV in cities in the Yellow River Basin, and explored the spatial heterogeneity (Figure 5). The results show that although the spatial distribution of the regional value of the Yellow River Basin did not change significantly during the study period, the spatial distribution pattern was obvious, due to differences in the land-use structure and geographic regions. Moran’s I test was performed on ESV, and the results showed that the estimated value changed slightly with time (0.370–0.381, *p* < 0.01), indicating that there is a significant spatial autocorrelation of urban ESV in the Yellow River Basin. From 1995 to 2018, the ESV spatial agglomeration pattern Lisa diagram shows that the Z-score was between 3.034–3.338 (*p* < 0.01), which means that ESV has a significant spatial agglomeration pattern during 1995–2018. Figure 4 shows the spatial distribution and distribution of high and low values in 1995 and 2018. The ESV performance of the Yellow River Basin is higher in the upper reaches than in the middle and lower reaches. The high ESV areas are located in Ordos, Bayanzhuoer, Hohhot in Inner Mongolia, Yulin in Shaanxi and Jiuquan in Gansu, mainly due to their vast land landscape types. It is also obvious that the cities in Henan and Shandong provinces in the lower Yellow River Basin have lower ESV values. This is mainly limited by its smaller land landscape type, the more developed urbanization development in the middle and lower reaches, and the interference of human activities in the landscape.

### 3.4. Coupling and Coordination Relationship between Urbanization and ESs in the Yellow River Basin

#### 3.4.1. The Overall Situation of the Coupling and Coordination Degree of Cities in the Yellow River Basin

After determining the comprehensive evaluation indicators of the two systems, we used the coupling coordination model introduced above to measure the degree of coupling and coordination D_it_**,** and the state of coupling coordination among the entire Yellow River Basin, the upper and middle reaches and the downstream cities from 1995 to 2018 (Table 5). It was found that the coupling and coordination between ESV and urbanization systems gradually improved during 1995–2018, from 0.287 to 0.386, but the overall level was low; the level of coupling and coordination transitioned from a severe imbalance to a mild imbalance. The coupling and coordination subtype changes were as follows: 1995–2000 showed a lagging urbanization, and 2005–2018 showed the simultaneous development of a mild imbalance between the two. This shows that at different stages of urban development, there are differences in the intensity and coordination of the interaction between urbanization and ESV, and the development trends are also different.

From the comparison between the upper and middle reaches of the sub-basin and the downstream, it was found that from 1995 to 2018, the overall degree of coupling and coordination of the upper and middle reaches was higher than that of the downstream area. The development of the upper and middle reaches experienced a lagging transition from mildly dysfunctional urbanization to a lagging development of mild dysregulation, while the overall downstream area experienced lagging development from a severely dyssynchronous transition to a mildly dysfunctional ESV. In the upper and middle reaches, which are in inland areas, due to the special topography and geomorphology, the development of urbanization is restricted, while the higher level of urbanization in the downstream areas imposes stronger constraints on the fragile ecological environment. This shows that the complexity of the performance of the urban coupling coordination degree within the Yellow River Basin, the coexistence of the economic quality improvement and environmental pressure pose serious challenges to the high-quality development of the Yellow River Basin.

#### 3.4.2. Spatio-Temporal Heterogeneity of Coupling and Coordination Degree of Cities in the Yellow River

**Figure 6:**
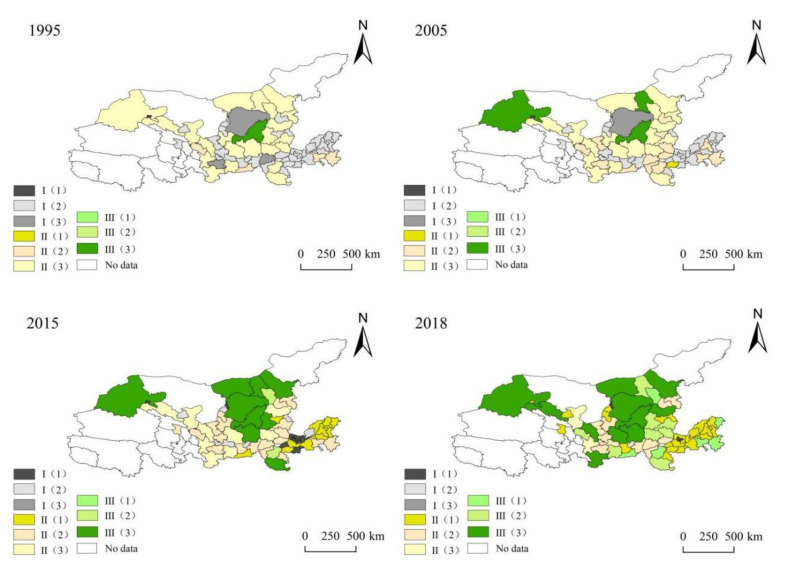
Spatio-temporal differentiation characteristics of urban coupling coordination degree in the Yellow River Basin, China.

This paper selected 1995, 2005, 2015 and 2018 as the cross-cutting years to analyze the spatial distribution of the coupling and coordination relationships among 63 prefecture-level cities in the Yellow River Basin (Figure 6). In general, the urban coupling degree of the Yellow River Basin showed an overall improvement trend, but the level of coupling coordination degree was still low and the spatial distribution difference was obvious. The high-value areas of the coupling coordination degree appear in the high-value areas of the northern ESV in the upper and middle reaches, and the low-value areas are distributed in cities in Shandong, Henan and other regions with relatively high urbanization levels in the middle reaches of the basin. The degree of development of ESV and urbanization in different regions is uneven, which poses a challenge for realizing the high-quality, coordinated development of regional urbanization and ESV and promoting the overall high-quality development of the Yellow River Basin.

In 1995, the degree of coupling and coordination of the Yellow River Basin showed three variations: 56.90% of the urban development was severely maladjusted, followed by 41.20% of the urban development in a mildly maladjusted co-loss type, and the cities with barely coordinated development accounted for only 1.59% of the total. The main manifestation of the mild disorder is the lagging urbanization type, and the main subtype of the severe disorder is the synchronous type, which was mainly caused by the backward economic development of the Yellow River Basin during that period. In particular, the industrialization structure of the heavy chemical industry during this period had obvious characteristics: the use of resources was relatively extensive; the consumption of resources and energy was large; and the high pollution, high energy consumption and high emissions in some areas caused great damage to the ecological environment [60].

In 2005, the degree of coupling and coordination in the entire Yellow River Basin was improved, compared with 1995, but the overall performance situation was similar to that in 1995, with 42.60% of the cities still in a serious state of imbalance. The overall performance showed two main coupling subtypes: synchronous type and lagging urbanization. The severely maladjusted cities are mainly in Shandong and Henan, as well as the following: Xianyang, Weinan, and Tongchuan in Guanzhong of Shaanxi; Jiayuguan, Jinchang, and Pingliang in Gansu; and Yinchuan, Shizuishan and Guyuan in Ningxia. Most of these areas are resource-based cities.

During the period 2005–2015, the overall coupling and coordination of the Yellow River Basin was significantly improved. The types of light coupling and coordination increased by 12.70%, and the number of cities with severe imbalances dropped sharply by 22.00%. In addition, the mild disorder subtype transformed from the mild disorder urbanization lagging dominance to the mild disorder synchronization type (30.10%) as the dominant form. This shows that the urbanization level and ES of the Yellow River Basin underwent significant changes at this stage. In 2018, with the improvement of the overall environment and urbanization level, the coordinated dispatch of ESV and urbanization in cities in the Yellow River Basin increased, with lightly coupled and coordinated cities reaching 39.68%, and the overall coupling and coordination degree subtype showed a trend of lagging ES. This shows that the gap in the level of urbanization in the entire study area gradually narrowed, and the ecological environment tended to deteriorate.

### 3.5. Factors Influencing the Degree of Coupling and Coordination between ESs and Urbanization

#### 3.5.1. Identification of Dominant Variables of Driving Factors

We used ArcGIS 10.4 to classify each element by the natural breakpoint method and calculated the influence of each element on the degree of coupling and coordination (Table 6). Based on the detection results, combined with the factor detection q value and significance test, the identification and construction of dominant factors were realized. Through the analysis of the detection results (Table 6), it was found that the *p*-values of the eight factors all reached a significance level of 1%, indicating that the coordinated development of urbanization and ESV is the result of the interaction of various elements with various types of driving forces. In addition, considering the explanatory power of sorting the q value, combined with the OLS regression results, we screened the variables for multicollinearity. Finally, GDP, environmental regulations (Env), technological level (Tech), road area (Road), proportion of forest land (For) and temperature (Tem) were selected to further discuss the spatial heterogeneity of influencing factors.

#### 3.5.2. Comparison of Influencing Factors Based on OLS-GWR Model

In order to further explore the spatial heterogeneity of the dominant factors affecting the degree of coupling coordination, this paper introduces geographically weighted regression for analysis and research. An important prerequisite for geographic weighted regression analysis is that the dependent variables have strong spatial autocorrelation. Therefore, this paper used Geo-da software to conduct Moran’s I test on the degree of coupling and coordination between ESs and urbanization. From the test results in Table 7, it can be found that from 1995 to 2018, the ESs and urbanization Moran’s I of the 63 sample cities were both positive at the 1% significance level. This shows that the coupling and coordination degree of urban ESs and urbanization development in the Yellow River Basin has obvious spatial spillover effects, so it is necessary to choose the GWR model to analyze the influencing factors of the coupling and coordination degree of the two.

Taking the sample of cities in the Yellow River Basin in 2018 as an example, ArcGIS 10.4 was used to construct OLS and GWR models, where the dependent variable is the degree of coupling and coordination between ESV and urbanization, and the independent variables are GDP, Env, Tech, Road, For and Tem. The regression results of the two types of models are shown in Table 8.

According to the research conclusion of Brundson [61], when the difference between the AICc value obtained in GWR and the AICc value in the OLS fitting result is greater than 3, it can be considered that the GWR model can better simulate the data. It can be seen from Table 8 that the application of the GWR model is more effective. In addition, the R^2^ of the GWR model and the adjusted R^2^ are 0.841 and 0.773, respectively, which once again shows that the GWR model has stronger explanatory power.

#### 3.5.3. The Spatial Differentiation Characteristics of the Influencing Factors of Coupling Coordination Degree

**Figure 7:**
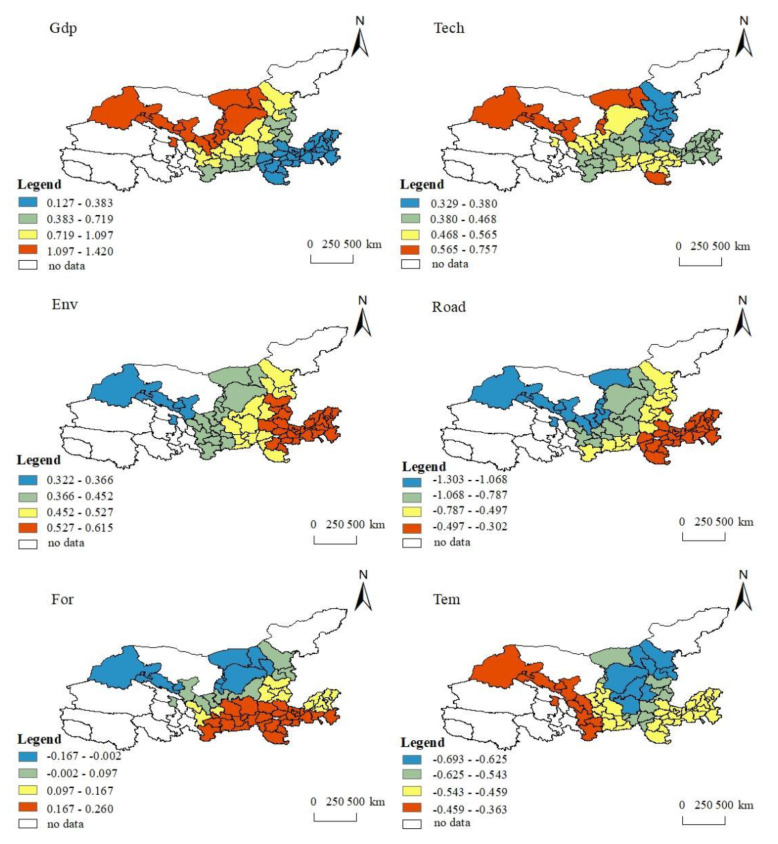
Spatial heterogeneity of the influencing factors of the coupling and coordination degree of urban ESV and urbanization in the Yellow River Basin, China.

Based on the above analysis, it can be considered that it is necessary to use the GWR model to investigate the different characteristics of the influencing factors in the urban spatial distribution of the study area. The Yellow River Basin basically covers the three major urban areas in the east, middle and west of China. The natural endowments and economic development of different cities are quite different. Therefore, the classification method of natural optimal break points was used to visualize the regression coefficients of various influencing factors (Figure 7), and further analysis of the differences between the various influencing factors of the coupling and coordination of ESV and urbanization at different spatial geographic unit locations was conducted.

(1) Regarding economic growth (GDP), from the perspective of the overall spatial distribution of driving characteristics, the driving intensity showed a belt-like decrease from upstream to mid-downstream. This shows that with the improvement of economic development and comprehensive strength, the attention and investment in ecological environmental protection and governance of cities increased, which effectively promoted the coordinated and coupled development of new urbanization and ESV. (2) At the level of science and technology (Tech), the overall driving distribution was from downstream to upstream, accompanied south to north, and the ring-shaped radiation gradually became stronger. This means that cities in the upper and middle reaches of the Yellow River should further increase investment in science and technology, continuously improve the level of innovation-driven development and strengthen ecological safety. (3) For environmental regulations (Env), the regression coefficients of environmental regulations to the degree of coupling coordination within the entire Yellow River Basin were all positive, and this positive impact gradually increased from upstream to downstream. (4) The regression coefficients of urban road construction were all negative in the entire watershed. From the perspective of spatial distribution characteristics, the negative effect in the downstream to upstream areas gradually increased. This shows that under the relatively special ecological environment of the Yellow River Basin, while strengthening urban infrastructure construction, attention should be paid to the management of the ecological environment, especially in the upper and middle reaches. (5) For the proportion of forest land (For), from the perspective of driving spatial distribution, the overall Yellow River Basin urban agglomeration was significantly positively driven by the proportion of forest land. From the perspective of the spatial distribution trend of driving strength, the positive effect of forest land on cities in the Yellow River Basin from north to south gradually increased. (6) For the temperature (Tem), from the perspective of the spatial distribution, the overall temperature of the urban agglomeration in the Yellow River Basin had a relatively obvious negative effect on the degree of coupling coordination. In terms of the spatial distribution of driving strength, from the southwest to the northeastern cities, from upstream to downstream, the negative driving force of the temperature-coupling coordination degree increased gradually outward, showing obvious radiation distribution characteristics.

## 4. Discussion

### 4.1. Changes in Urbanization, Land Use and ESV

This paper aimed to reveal the coupling mechanism between urbanization and ESV and its key driving factors. First, we established a four-dimensional urbanization measurement system of economy, society, population and space, which can better reflect the comprehensive level of urbanization than the single indicator used in the past [27]; it is also conducive to the analysis of endogenous influencing factors that affect the degree of coupling and coordination. Second, we quantified the changes in land-use types in the urban agglomerations of the Yellow River Basin and found that during the study period, the land-use types changed significantly. Specifically, grassland and farmland have always been the main types of land use in the Yellow River Basin, but the area of them showed a downward trend. A large amount of grassland was converted into forest land and partly into construction land [62]. The areas of forests, wetlands, water and construction land increased. Among them, construction land increased by the largest proportion, reaching 61.10% (22.66 × 10^5^ ha). This is consistent with the previous research results [45], and it also poses challenges to the ecological environment protection and land management of the Yellow River. In addition, the increase in waters and wetlands is mainly attributed to two reasons. On the one hand, it is due to unutilized conversion, which is consistent with the research of related scholars [62]; on the other hand, the number and area of newly built lakes and reservoirs increased to 57 new lakes and reservoirs in the basin, and 55 that expanded in area [63]. The increase in area is consistent with the trend of annual precipitation. According to the Yellow River Net’s 2018 Water Resources Bulletin, the average precipitation in 2018 is 551.6mm, which is 23.4% larger than the average value from 1956 to 2000, and the annual runoff measured by the hydrological station on the mainstream of the Yellow River in 2018 is 5.8–46.5% larger than the average value from 1956 to 2000 (http://www.yrcc.gov.cn/, accessed on 8 July 2021).

We further used the equivalent factor method to analyze the changing characteristics of ESV. The results show that ESV in most areas of the Yellow River Basin is on the rise, and the overall improvement is greater than the deterioration. This shows that the Yellow River Basin governance has obtained certain achievements, but there is still pressure for ecosystem protection and restoration. Since 2000, China has implemented numerous ecological restoration projects, such as returning farmland to forests and grasslands, and protecting natural shelter forests. These measures have led to the continuous increase in vegetation area and also actively promoted the transformation of land landscape patterns [47,48]. At the same time, these also enable the national ecological economy and green development concepts to be realized in the process of urbanization. The ESV of forest, water and wetland showed different growth rates, which offset the decline in ESV accompanying the increase in urbanization, while the ESV of grassland and farmland declined; key protection and restoration should be carried out. Therefore, forest land and water bodies have high ESV per unit area, which is of great significance for promoting the operation of the overall regional ecosystem and maintaining regional ecological services. In the future regional land use management of the Yellow River Basin, priority should be given to areas with high estimated value and sensitive areas vulnerable to urbanization [25,27].

Although the Yellow River Basin has achieved good results in ecological management and restoration in recent decades, the regional biodiversity and ecosystem functions still need to be improved. Some plantations have a single species composition, and the proportion of trees, shrubs and grasses is uneven [64]. For this reason, it is recommended to further strengthen the protection and restoration of biodiversity in the ecological engineering of the Yellow River Basin by advocating solutions based on nature and shifting from engineering restoration to focusing on protection and natural restoration [65]. It is not appropriate to simply exclude human activities, but it is necessary to find a balance between human activities and biodiversity conservation.

### 4.2. The State of Coupling and Coordination between Urbanization and ESV

As a bridge that couples natural and social processes, the practicality of ESs can provide quantitative information on relevant policies for urban planning and land resource management through the evaluation of the coupling and coordination relationship between regional urbanization development and ESV, such as helping to determine priority management issues and adjusting the direction of the plan [66].

This study analyzed the overall situation of the coupling coordination degree of the Yellow River Basin and explored its spatial differentiation. During the study period, the degree of coupling and coordination between urbanization and ESV in the Yellow River Basin transitioned from severe imbalance to mild coupling, coordination and mild imbalance. This proves that the interaction between ESV and urbanization in the Yellow River Basin is very close, and the two have formed a close dependency relationship. After experiencing an extensive economic growth model, people paid more attention to the protection of the ecological environment of the Yellow River Basin and to promoting urbanization by improving resource utilization efficiency and people’s lifestyles, so the degree of coordination continues to rise. According to the coupling index between average ESV and urbanization, the coupling subtype changed from urbanization lagging type to simultaneous development and ESV lagging. This proves that the low conflict and potential crisis are the main relationships between them, and with the development of urbanization, the growth of ESV lags behind the degree of urbanization. The rapid urbanization area occupies a part of the cultivated land area, which has a coercive effect on the fragile environment [8]. Therefore, in the process of urbanization, we should pay attention to the coordination between rational land use and economic society, strengthen the sustainable agriculture and food diversity transformation, improve the resilience of local and global agricultural systems, and promote mitigation of and adaptation to climate change. At the same time, sustainable agriculture can also provide a habitat for biodiversity, reduce the pressure on forests and other biodiversity ecosystems and maintain the health and well-being of the population [67,68].

In terms of spatial distribution, there are obvious regional differences, with ESV lagging mainly in the lower reaches, and urbanization lagging mainly in the upper and middle reaches; the overall characteristics of natural basic divisions and administrative boundaries are presented. The severely maladjusted cities are mainly the cities in Shandong and Henan in the lower reaches of the basin, and most of these are resource-based cities. Due to the extensive use of resources, the single industrial structure, the lack of support from alternative industries, and the high pressure of industrial transformation and upgrading, the economic and social development has a serious negative impact on the ecological environment, which also leads to a relatively low degree of coupling and coordination [69,70].

Due to the special ecological background and economic development characteristics, the degree of coupling and coordination between the urbanization of the Yellow River Basin and ESV can easily form a co-destructive development, that is, dual stress effects [71]. The economic development of the upper reaches of the Yellow River lags behind, and the terrain is dominated by mountains and hills, facing the problems of ecosystem degradation and reduced water conservation functions; the Loess Plateau in the middle reaches has serious soil erosion; and the urbanization level of the downstream urban agglomerations is outstanding, so the ecological flow is low, and the wetlands are shrinking [72]. In addition to the differences caused by factor endowments, it should be noted that the losses suffered by the upstream area to protect the ecological environment have further aggravated the differentiation of economic space; downstream residents, as the beneficiaries of the improvement of the ecological environment, should appropriately bear the responsibility of ecological protection [48], and establish and perfect an adaptation compensation mechanism for the coordinated development of regional coupling.

In order to alleviate the ecological problems caused by rapid economic development, the Chinese government has adopted a series of measures [16,48]. The assessment of resources, the environmental carrying capacity, and suitable land forms the basis of national land planning. ESs, including biodiversity, soil and water conservation, soil conservation and wind and sand fixation, are included in China’s current land planning policies [8]. However, further incorporating the type of coupled and coordinated development, national ecological function zoning and resource carrying capacity into the national land plan is a direction worth considering in the future.

### 4.3. Key Factors of Coupling Mechanism

Based on the scientific evaluation of the coupling and coordination state of urbanization and ESV, this paper further analyzed the spatial differentiation of influencing factors through geographic detectors and GWR models embedded with geographic location information and gave corresponding policy recommendations. The study found that the factors that affect the coupling and coordination relationship are embodied in different aspects and present different spatial differentiation characteristics.

Economic growth, environmental regulation and technological innovation show obvious positive driving characteristics. For areas with developed urbanization and areas with relatively low ESV, there is an urgent need to increase the intensity of environmental regulations, pay attention to the political spillover effects of environmental regulations and to not blindly pursue rapid urbanization while ignoring environmental management and ecological safety protection. For cities in the upper and middle reaches of the Yellow River, consideration should be given to further increasing investment in science and technology; promoting the growth of regional economic quality through talent accumulation, technological innovation and institutional innovation; and, on this basis, achieving economic integration and innovation to promote comprehensive economic development in order to provide a material guarantee for the improvement of the development level of the ecosystem and the coupling and coordination of urbanization subsystems.

Forests also reflect the positive driving effect of high quality. Vegetation is an important part of the ecosystem. It is sensitive to changes in the ecological environment and plays a pivotal role in the urban ecosystem [73,74]. As a genetic preservation of biodiversity, forest ecosystems have outstanding performance in many aspects of climate and environmental regulation and soil hydrology improvement. The increase in species diversity and community complexity will directly lead to the improvement of ESs [75]. Therefore, in the process of urbanization, land use and urban forest construction should be planned as a whole, and ecosystem or landscape methods should be applied to reduce the pressure of land on forests and other natural ecosystems, avoid further loss of habitats and create conditions to restore more habitats, thereby reducing the risk of species extinction. This will protect and increase the income and sources of nutrition for people who depend on forest ecosystems for their livelihoods. Many cultural connections with forest species and landscapes will be protected, while also contributing to health and well-being [76,77,78].

In addition, temperature, as an important natural factor, causes changes in the growth environment of vegetation, the intensity of vegetation activities and ecosystem service functions [79,80]. However, the driving factors of vegetation changes in the environment of urban agglomerations are more complex, being not only affected by natural factors, but also by environmental changes caused by urbanization, especially the heat island effect in urban expansion, which will also lead to changes in the urban ecological environment [81]. Therefore, not only should the decline in ESV caused by forest loss in the process of urban expansion be alleviated, but also the “cold island effect” role played by urban green space should be emphasized to reduce the urban heat island effect [41].

Taking the area of road construction as the intensity of infrastructure construction, it was found that the strengthening of urban infrastructure led to a decrease in the degree of coupling and coordination. This shows that blindly pursuing urban construction and ignoring the protection of urban ecosystems will lead to unilateral coercion of the system. Therefore, urban planners should pay attention to the high-quality coordination effect of the smart city and urban forest landscape pattern based on the information and technology of the two systems [82]. Through the process of environmental assessment and large-scale zoning covering biological diversity, in infrastructure investment planning and development, such as transportation system design and management, the consideration of biological diversity is reflected to avoid the most vulnerable of biological diversity. Taking measures to protect ecological connectivity, such as through overpasses, underground passages and green infrastructure, promotes the transition to a more sustainable urbanization model [6].

In addition, the advancement of new-type urbanization and the high-quality development strategy of the Yellow River Basin will inevitably promote the rapid connection of material, energy and information among regions [27]. Cities in the Yellow River Basin should take this as an opportunity to establish a linkage mechanism, relying on the corridors that have been formed in the urban agglomeration to improve the functional structure of each city, improve the spatial allocation efficiency of land resources and realize the high-quality coordinated development of ESV and urbanization as soon as possible.

### 4.4. Limitations and Implications

This research deeply explored the spatial differentiation characteristics of the driving mechanism of the coupling and coordination relationship between urbanization and ESV, and emphasized making full use of the positive spillover effects between regions to create a sustainable network management pattern with coordinated planning and systematic governance across regions. It is suggested that the types of coupled and coordinated development, national ecological function zoning and resource carrying capacity should be further incorporated into the national land plan. However, this study has several limitations, which can provide a reference for future research. First, in this study, we set the equivalent factor of construction land to 0. Although many studies have the perspective that the ESs provided by construction land are of no value [83], some studies propose that construction land can provide some ESs, such as entertainment, tourism and culture [84]. Taking into account that in the process of urbanization, the transition from natural ecosystems to semi-artificial ecosystems and artificial ecosystems will inevitably damage the ecosystem, as well as the hysteresis effect of ESs, we propose to set the construction land to 0. Second, due to the complexity of human activities, further analysis of endogenous influencing factors is also a very important aspect in future exploration. How to coordinate internal and external factors to manage the ecological network and spatial control of urban agglomerations is of great significance. Third, due to the lack of a small amount of data at the urbanization level of large regions, individual regions cannot be included in the evaluation system, so exploring a more scientific and comprehensive urbanization measurement method will also be an important and difficult area of research in the future. In addition, in terms of ESV estimation, more scientific, rigorous and accurate methods should be explored. Especially in order to measure the spatial transformation and scale effect of ESV, a more systematic and in-depth dynamic evaluation is urgently needed to reflect the continuous annual and quarterly dynamic changes in ESV.

## 5. Conclusions

This paper discussed urbanization from multiple perspectives, assessed the changes in urbanization and ESV in the Yellow River Basin as well as their temporal and spatial relationships during the study period, and deeply analyzed the driving mechanism that affects the coupling and coordination relationship between urbanization and ESV. This provides a scientific basis for the high-quality development strategy for the urban agglomeration in the Yellow River Basin.

1. From 1995 to 2018, the urbanization level of the urban agglomerations in the Yellow River Basin was continuously improved, and the development characteristics changed from the initial urbanization stage to an intermediate stage characterized by social urbanization and economic development. During this period, the ESV of the Yellow River Basin was greatly improved, mainly due to the overall increase in the value of forests, water and wetlands, which offset the expansion of construction land and the decrease in ESV of farmland and grassland. Overall, regulation services occupied a dominant position. Although the spatial distribution of regional values in the Yellow River Basin did not change significantly in the study period, the spatial distribution pattern was obvious, due to differences in the land-use structure and geographic regions.
2. From 1995 to 2018, the degree of coupling and coordination improved significantly. Mildly coupled coordination gradually increased, severe imbalance types tended to disappear and coupling subtypes developed from lagging urbanization to ESV backward and synchronized types. However, overall, there was still a low-level coupling and coordination process, and there were obvious regional differences, showing the emergence of boundaries between physical geographical conditions and administrative divisions. Especially in the lower reaches of Henan, Shandong, other regions and most of the resource-based cities in Central China, the degree of coupling was significantly lower. Therefore, we should be guided by high-quality coordination, divide functional areas for different levels of coordination and implement different strategies.
3. In addition, factors such as economic growth, technological development, environmental regulations and the proportion of forest land had positive and belt-like alienation characteristics for the coupling and coordination of the two, and infrastructure and temperature showed negative driving characteristics. Therefore, the Yellow River Basin should uphold the characteristics of basin integrity and differentiation, comprehensively coordinate various driving factors, create regional coordinated planning and coordinated governance, and promote the high-quality development of the coordinated relationship between urbanization and ESV.

## Figures and Tables

**Table 1:** Comprehensive evaluation index system for urbanization in the Yellow River Basin, China.

First-Grade Indicator	Weight	Basic Grade Indicator	Unit	Weight
Demographic urbanization	0.114	Population density	persons/km^2^	0.046
		Urban population density	persons/km^2^	0.047
		Percentage of non-agricultural population	(%)	0.021
Landscape urbanization	0.251	Percentage of built-up areas in the total land area	%	0.070
		Number of urban areas per 10,000 people	km^2^	0.079
		Paved road area per capita	m^2^	0.037
		Green area per capita	m^2^/person	0.052
		Green coverage rate of built-up area	%	0.013
Economic urbanization	0.398	GDP per capita	CNY	0.064
		The proportion of secondary and tertiary industries in GDP	%	0.005
		Gross industrial output value above designated size	10^4^ CNY	0.108
		Total fixed asset investment	10^4^ CNY	0.092
		Local financial revenue per capita	CNY	0.085
		Average salary of employees	CNY	0.043
Social urbanization	0.273	Total retail sales of consumer goods per capita	CNY	0.075
		Number of primary and middle school students	10^4^ persons	0.038
		Public library collections per capita	volume	0.032
		Beds in health care industry per 10,000 people	bed	0.015
		Internet users per 10,000 people	persons	0.077

Note: GDP stands for gross domestic product; CNY stands for Chinese Yuan and is the standard currency symbol.

**Table 2:** Dynamic factors of coupling and coordination degree in the Yellow River Basin, China.

	Variables	Symbol	Variable Description	Unit
Economic factors	Economic Growth	GDP	GDP Growth Rate	%
	Industrial structure	Ind	The industrial structure upgrade is obtained by the weighted square: Ind = $\sum_{1}^{3}$S_n_ × n, 1 ≤ n ≤ 3, where n represents the proportion of the n-th industrial output value. In terms of economic meaning, the closer the value of Ind is to 1, the lower the level of the industrial structure of the place, and the closer the value of Ind is to 3, the higher the level of industrial structure of the place [53].	
	Educational investment	Edu	Education expenditure as a proportion of fiscal expenditure	%
	Government capacity	Gov	Regional fiscal expenditure as a percentage of GDP	%
Social factors	Environmental regulation	Env	The entropy method is used to combine industrial wastewater discharge compliance rate, SO_2_ removal rate and solid waste comprehensive utilization rate into one indicator to indicate the strength of environmental regulations [54]. Env = ∑_ij_ = G_ij_ × W_ij_ W_ij_ refers to the weight of index j in city i, G_ij_ represents the standardized value of index j in city i, Env is the environmental regulation in city i, which is the sum of all the indicators’ regulation indexes.	
	Technological innovation	Tech	Number of granted technology patents	pieces
	Infrastructure	Road	Actual urban road area at the end of the year	10^4^ km^2^
	Total population	Pop	The total population of the city at the end of the year	10^4^ person
Natural factors	Temperature	Tem	The annual average temperature	℃
	Precipitation	Pre	Average annual precipitation	mm
	Terrain relief	Ter	Altitude difference between the highest and lowest points	m
	Percentage of woodland	For	Woodland land type area as a proportion of total area	%

**Table 3:** Judgment criteria for coupling relationship.

Coupling Coordination Type	Coordinated Development	Contrast Relationship	Subtype
Severe imbalance (I)	0 < D ≤ 0.3	λ_U_ − λ_E_ > 0.1	Severely maladjusted ESV hysteresis type (1)
		|λ_E_ − λ_U_| ≤ 0.1	Severely maladjusted synchronous type (2)
		λ_E_ − λ_U_ > 0.1	Severely unbalanced urbanization lagging type (3)
Mild maladjustment (II)	0.3 < D ≤ 0.4	λ_U_ − λ_E_ > 0.1	Mild dysregulation ESV hysteresis type (1)
		|λ_E_ − λ_U_| ≤ 0.1	Mild imbalance and co-loss type (2)
		λ_E_ − λ_U_ > 0.1	Mild imbalance and lagging urbanization (3)
Mild coupling coordination (III)	0.4 < D ≤ 0.7	λ_U_ − λ_E_ > 0.1	Slightly coupled coordinated ESV hysteresis type (1)
		|λ_E_ − λ_U_| ≤ 0.1	Lightly coupled, coordinated and synchronized type (2)
		λ_E_ − λ_U_ > 0.1	Slightly coupled and coordinated urbanization lagging type (3)
High-quality coupling and coordination (IV)	D > 0.7	λ_U_ − λ_E_ > 0.1	High-quality coupling and coordination ESV hysteresis type (1)
		|λ_E_ − λ_U_| ≤ 0.1	High-quality coupling, coordination and synchronization (2)
		λ_E_ − λ_U_ > 0.1	High-quality coupling and coordinated urbanization lagging type (3)

**Table 4:** Ecosystem service value (ESV) of various landscape types in the Yellow River Basin (China) in 1995 and 2018.

	ESV (CNY 10^9^)				Change Value (10^9^ CNY)/Change Rate (%)			
Year	1995	2005	2015	2018	1995/2005	2005/2015	2015/18	1995/2018
Farmland	150.52	151.07	148.96	146.82	0.55/0.35	−2.11/−1.40	−2.14/−1.44	−3.70/−2.46
Forestland	152.38	160.95	161.18	161.39	8.58/5.63	0.22/0.14	0.22/0.13	9.02/5.92
Grassland	418.55	403.81	398.43	398.25	−14.74/−3.52	−5.38/−1.33	−0.18/−0.05	−20.31/−4.85
Water bodies	13.87	13.41	15.67	16.97	−0.46/−3.30	2.25/16.79	1.31/8.33	3.10/22.35
Wetland	99.87	111.95	107.33	121.04	12.08/12.10	−4.62/−4.13	13.71/12.77	21.16/21.19
unused land	7.95	7.94	7.91	7.76	−0.01/−0.13	−0.02/−0.26	−0.15/−1.92	−0.18/−2.31
Total	843.14	849.13	839.48	852.23	5.99/0.17	−9.66/−1.14	12.75/1.52	9.09/1.08

**Table 5:** Yellow River Basin City ESV and urbanization coupling coordination degree.

	Full Sample		Upstream and Midstream		Downstream	
Year	D_it_	Basic Type	D_it_	Basic Type	D_it_	Basic Type
1995	0.287	Severely unbalanced urbanization lagging type	0.302	Mild imbalance and lagging urbanization	0.250	Severely maladjusted synchronous type
2000	0.293	Severely unbalanced urbanization lagging type	0.306	Mild imbalance and lagging urbanization	0.263	Severely maladjusted synchronous type
2005	0.311	Mild imbalance and co-loss type	0.322	Mild imbalance and lagging urbanization	0.283	Severely maladjusted synchronous type
2010	0.342	Mild imbalance and co-loss type	0.352	Mild imbalance and lagging urbanization	0.318	Mild imbalance and co-loss type
2015	0.350	Mild imbalance and co-loss type	0.359	Mild imbalance and lagging urbanization	0.328	Mild dysregulation ESV hysteresis type
2018	0.386	Mild imbalance and co-loss type	0.396	Mild imbalance and co-loss type	0.361	Mild dysregulation ESV hysteresis type

**Table 6:** Geographical detector detection results.

Impact Factors	GDP	Ind	Gov	Edu	Tech	Env	Road	Pop	Pre	Tem	Ter	For
*q* value	0.677	0.320	0.170	0.139	0.578	0.625	0.678	0.435	0.185	0.517	0.051	0.459
*p* value	0.000	0.000	0.013	0.035	0.000	0.000	0.000	0.000	0.024	0.000	0.368	0.000

Note: q value is the explanatory power of variable x to Y; *p* value represents the probability.

**Table 7:** Moran’ I of the coupling and coordination degree of urbanization and ecosystem services in the Yellow River Basin, China.

Year	1995	2000	2005	2010	2015	2018
Moran’s I	0.310 ***	0.253 ***	0.261 ***	0.237 ***	0.216 ***	0.186 ***
Z-score	3.937	3.324	3.359	3.106	2.859	2.510

Note: *p* value represents the probability; Z score represents the multiple of the standard deviation; *** represents the significance at 1% level.

**Table 8:** Comparison of estimation results between OLS (Ordinary Least Squares) and GWR (Geographically Weighted Regression) models.

OLS Model					GWR Model				
	Coefficient	*t* Value	*p* Value	VIF	Mean	Std	Min	Med	Max
GDP	0.496	1.839	0.066	1.068	0.710	0.411	0.127	0.634	1.420
Tech	0.485	2.587	0.010	2.923	0.491	0.099	0.329	0.458	0.757
Env	0.478	4.783	0.000	2.300	0.496	0.086	0.322	0.512	0.615
Road	−0.538	−2.737	0.006	2.320	−0.683	0.339	−1.303	−0.569	−0.302
For	0.191	2.112	0.035	1.119	0.126	0.101	−0.167	0.155	0.260
Tem	−0.518	−4.563	0.000	1.595	−0.521	0.082	−0.693	−0.520	−0.363
R^2^	0.574				0.841				
Adj. R^2^	0.528				0.773				
AICc	143.766				151.628				

Note: VIF is the coefficient of variance expansion. If all VIFs are less than 10, it means that the model has no multicollinearity problem; min, max, std and med represent the minimum, maximum, standard deviation and median of the estimated coefficients of the GWR model, respectively.

## Data Availability

The data presented in this study are available on request from the corresponding author. The data are not publicly available, as part of them are being used in other studies that have not yet been publicly published.
